# Impacts of Decaying Aromatic Plants on the Soil Microbial Community and on Tomato Seedling Growth and Metabolism: Suppression or Stimulation?

**DOI:** 10.3390/plants10091848

**Published:** 2021-09-06

**Authors:** Aggeliki Ainalidou, Foteini Bouzoukla, Urania Menkissoglu-Spiroudi, Despoina Vokou, Katerina Karamanoli

**Affiliations:** 1 Laboratory of Agricultural Chemistry, Faculty of Agriculture Forestry and Natural Environment, School of Agriculture, Aristotle University of Thessaloniki, 54124 Thessaloniki, Greece; ainalidoy_aggeliki@yahoo.gr (A.A.); vouzouklafoteini@gmail.com (F.B.); 2 Pesticide Science Laboratory, Faculty of Agriculture Forestry and Natural Environment, School of Agriculture, Aristotle University of Thessaloniki, 54124 Thessaloniki, Greece; rmenkis@auth.gr; 3 Department of Ecology, School of Biology, Aristotle University of Thessaloniki, 54124 Thessaloniki, Greece

**Keywords:** biostimulant, essential oil, herbicide, *Mentha piperita*, *Mentha spicata*, PLFA, priming, *Rosmarinus officinalis*, soil amendment, threonine

## Abstract

This study provides insight into changes in the features of tomato seedlings growing in soils enriched with spearmint, peppermint, or rosemary leaves and into changes in the microbial communities of these soils used as seedbeds; an organic amendment was also applied as a positive control. While the soil microbial community flourished in the presence of all three aromatic plants, tomato growth was inhibited or stimulated depending on the plant that was used. More specifically, phospholipid fatty acid (PLFA) analysis showed an increase in the total microbial biomass and in the biomass of all the groups examined, except for actinobacteria, and changes in the microbial community structure, with Gram-negative bacteria and fungi being favoured in the mint treatments, in which the microbial biomass was maximized. Seedlings from the rosemary treatment were entirely inhibited; they were at the open-cotyledon stage throughout the experiment. Seedlings from the mint treatments were the heaviest, longest, and had the highest chlorophyll content and photosynthetic yield. Metabolomic analysis showed metabolism enhancement associated with both growth and priming in seedlings from the mint treatments and disruption of metabolic pathways in those from the rosemary treatment. There is a great potential for applying these aromatic plants as soil amendments and as either biostimulants of plant growth or as herbicides.

## 1. Introduction

Demand for agricultural products has been continuously expanding in response to the rapidly growing human population and a constantly changing environment. To satisfy this demand, several strategies have been developed, including the extensive use of agrochemicals. While contributing to higher agricultural production, agrochemicals are associated with serious environmental impacts such as pollution, toxicity, low soil fertility, high disease incidence, reduced product quality or low efficacy, and heavy dependence on energy inputs [1], thus contributing to the current climate change. An approach to overcome the problems created by the heavy use of synthetic chemicals is to replace them with products of natural origin.

Use of environmentally friendly products as soil amendments, fertilizers, and phytoprotectants is a major goal in organic farming. Aromatic plants are candidate sources for such products. Because of their multifaceted biological activity and the important roles that they play in plant–plant and plant–microbe interactions [2,3,4], essential oils produced by these plants are recognized as valuable natural products for several uses. Among the activities that have been attributed to them, prominent are those related to microbial and plant growth, which are both inhibitory and stimulatory. Essential oils have gained much interest for their use in agriculture because of their (i) natural origin, making them less harmful to the environment than synthetic chemicals, (ii) volatility, implying less residue on the produce or in the environment after application, and (iii) composite nature, implying multiple mechanisms of action [5,6,7].

Regarding microbes, apart from the well-known inhibitory activity of essential oils against human, animal, and plant pathogens [8,9,10], promoting effects to microbial populations and associated soil and/or foliar metabolism have been found. For instance, it has been reported that essential oils increase soil respiration, which is an indicator of soil fertility [11,12,13,14], that epiphytic bacterial communities on aromatic plants are not less abundant or diverse than communities on non-aromatic plants [15,16], that specific bacterial strains are even able to grow on oregano glandular hairs [17], and that the sporulation of some fungi may become much higher in the presence of some essential oils or their constituents [10]. In addition, many bacteria are reported as having the ability to degrade essential oil ingredients [18], whereas most essential oils are reported as having only weak antimicrobial activity [19].

Regarding plants, many essential oil constituents exert inhibitory effects on seed germination and seedling growth, with most groups of oxygenated compounds (alcohols, aldehydes, ketones, or phenols) being considerably more active than hydrocarbons [20,21,22]. The compounds that are present may not behave in an additive way but may instead behave synergistically or even antagonistically [21]. Essential oils and their constituents have been tested in vitro against several weeds and crops, including tomato [23,24], with varying effects largely depending on the molecules involved and the target species. The latter shows selectivity of action, which is a highly desirable feature for agricultural applications. However, as microbial interference may change the structure of the active molecules and, hence, the plant–plant interactions expected from in vitro experiments, such results need to be verified in vivo.

Given the importance of soil conditions in maintaining sustainability and environmental quality [25] as well as the plant and microbial growth-related activities of essential oils, the performance of aromatic plants as soil amendments and alternative crop-stimulating/crop-protecting agents has been assessed. In this context, spearmint composts [26] or leaves [27,28] incorporated at different rates in the soil, stimulated the growth of tomato plants remarkably while also suppressing weed emergence, particularly of broadleaved species [26]. Other aromatic plants such as *Thymus citriodorus* were also found to exert a beneficial effect on tomato growth while successfully controlling root knot nematodes [29]. 

A study of the changes in the essential oils of spearmint, peppermint, and rosemary during the decay process of their green parts in the soil environment [30] showed a rapid decrease in the essential oil content for spearmint and peppermint but not for rosemary. Rapid changes were also observed in the composition of essential oils: the relative contribution of monoterpenoids in spearmint and peppermint dropped from about 90% initially to 45% and 20%, respectively, 60 days later, while sesquiterpenoids increased both in number and relative contribution in the same period. In contrast, rosemary oil remained almost unaltered for at least 90 days. Using the same aromatic plants and setting as in [30], here, we examine how the soil microbial community changes with time as aromatic plants decay and how they affect tomato growth, photosynthesis, and metabolism. Such a multidimensional assessment will enable us to gain further insight into how essential oils influence soil communities and soil processes and evaluate their potential to be used as soil amendments and biostimulants for crop growth. 

## 2. Results

### 2.1. Structure and Abundance of the Soil Microbial Community

**Figure 1:**
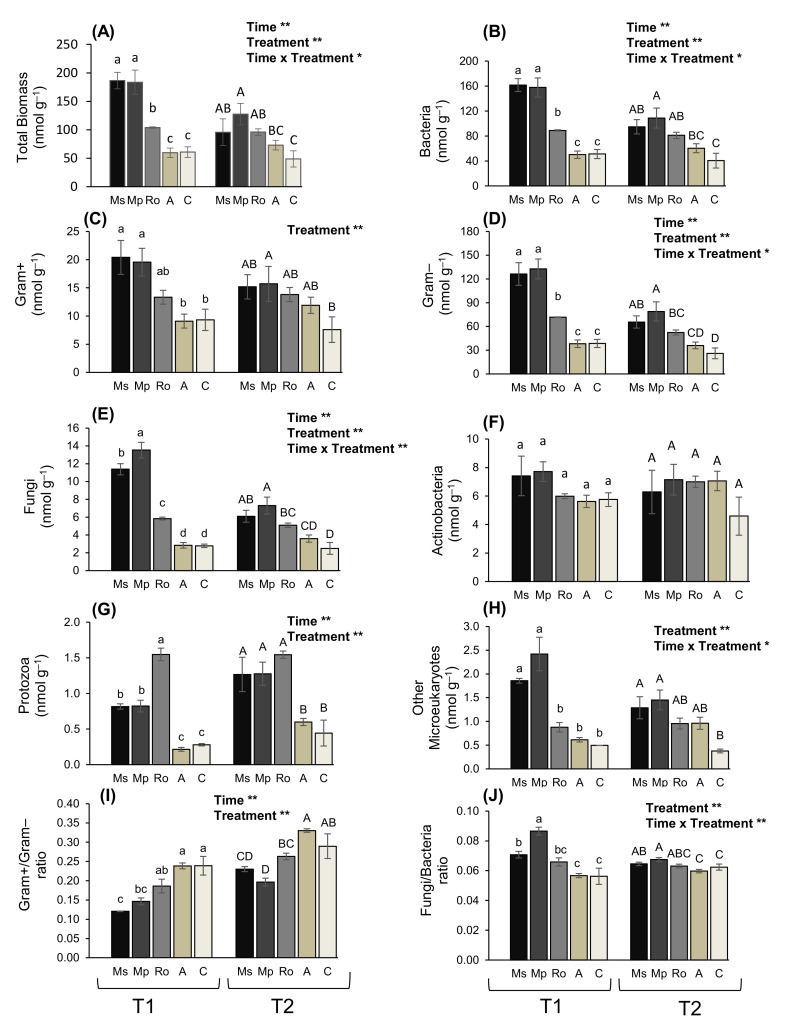
(**A**) Total microbial biomass and biomass of (**B**) bacteria, (**C**) Gram–positive bacteria, (**D**) Gram–negative bacteria, (**E**) fungi, (**F**) actinobacteria, (**G**) protozoa, (**H**) other microeukaryotes, and ratios (**I**) of fungi to bacteria and (**J**) of Gram–positive to Gram–negative bacteria, in soils treated with the aromatic plants *Mentha spicata* (Ms), *M. piperita* (Mp), and *Rosmarinus officinalis* (Ro) as well as in those treated with an organic amendment (**A**) and in control soil (**C**). Measurements were taken at T1 and T2, 28 and 56 days, respectively after the soil mixtures and the control were prepared. Values are means of three replicates ± SE. Results of the analysis (two-way ANOVA) are indicated on each graph (**: *p <* 0.01; *: *p <* 0.05). Different letters above bars (small for T1 and capital for T2) correspond to significant differences among treatments within each sampling time (Duncan’s multiple range test; *p <* 0.05); values between different times were not compared.

When using a soil amendment, a number of beneficial outcomes are expected on the crop growth and yield as well as on the functionality of soil communities. To investigate the impact of aromatic plants incorporated in the soil on microbial biomass and to further explore the induced structural and functional changes in the microbial communities, the treated soils were subjected to PLFA analysis. Overall, 28 and 26 fatty acid methyl-esters of microbial origin were detected at T1 and T2, respectively, excluding the internal standard of 19:0 (Tables S1 and S2). Among them, there were several methyl-esters of fatty acids that are considered indicators of specific microbial groups [31,32,33]. These are the following: 15:0, a-15:0, i-15:0, i-16:0, 17:0, a-17:0, and i-17:0 for Gram-positive bacteria; 11Me 18:1ω6, 16:1ω7c, 18:3ω6,9,12, and cy17:0 for Gram-negative bacteria; 18:2ω6,9 and 18:3ω3,6,9 for fungi; 10Me16:0, 10Me17:0, and 10Me18:0 for actinobacteria; 20:5ω3 and 20:4ω6 for protozoa; 22:0, 23:0, and 24:0 for other microeukaryotes. The remaining PLFAs may be derived from several sources: for instance, 18:2ω3,9*c*, 18:1ω9*c,* and 18:2ω6*t* are derived from both Gram-negative bacteria and fungi, 16:0 can be derived from bacteria and fungi, whereas 12:0, 14:0, 17:1, 18:0, and 20:0 are not regarded as indicators of any specific group. The sum of all the amounts for the identified lipids of microbial origin was used as a proxy of total microbial biomass (in nmol g^−1^). Total microbial biomass was affected by the treatment and sampling time (Figure 1A). Aromatic plants induced a significant increase in the total PLFA yield compared to the control, which persisted for the whole duration of the experiment; however, the values were lower at T2. The organic amendment treatment (A) had no effect on the soil microbial community. 

To explore which microbial group is affected when enriching the soil with aromatic plants, the changes in the biomass of the different microbial groups were examined (Figure 1B–H). Actinobacteria (Figure 1F) were affected neither by treatment nor by sampling time, whereas Gram-positive bacteria (Figure 1C) and other microeukaryotes (Figure 1H) were not affected by sampling time. For all the other examined groups (bacteria, fungi, Gram-negative bacteria, and protozoa), both the treatment and sampling time had significant effects. Bacteria was the most abundant group at both sampling times (T1 and T2), with their biomass being at least an order of magnitude higher than that of fungi. Similarly, Gram-negative bacteria had a biomass about an order of magnitude higher than Gram-positive bacteria. At T1 (after 28 days), in soils treated with aromatic plants, the bacterial and fungal biomass was higher compared to the control; it was also higher than in the organic amendment (A) samples. Similarly, a biomass that was higher than the control was also detected at T2 (after 56 days) in soils treated with aromatic plants. Leaving aside actinobacteria, and with one exception, all the microbial groups were the most abundant in the Ms and Mp treatments; however, values were not always significantly different from those of the Ro treatment; the exception was protozoa, the biomass of which was highest in the Ro treatment at T1. No significant effect was detected for any of the microbial groups in the soil samples of the organic amendment (A). At both sampling times, the ratio of fungi to bacteria was higher than the control in the Ms and Mp treatments (Figure 1J), while the ratio of Gram-positive to Gram-negative bacteria was lower in the same treatments (Figure 1I). With the exceptions mentioned above, the highest overall values for all the microbial groups were recorded in the Ms and Mp treatments at T1, showing that the microbial biomass was maximized early, four weeks from the start of the decomposition of the aromatic plants that were added in the soil. 

### 2.2. Plant Growth Responses of Tomato Seedlings

**Figure 2:**
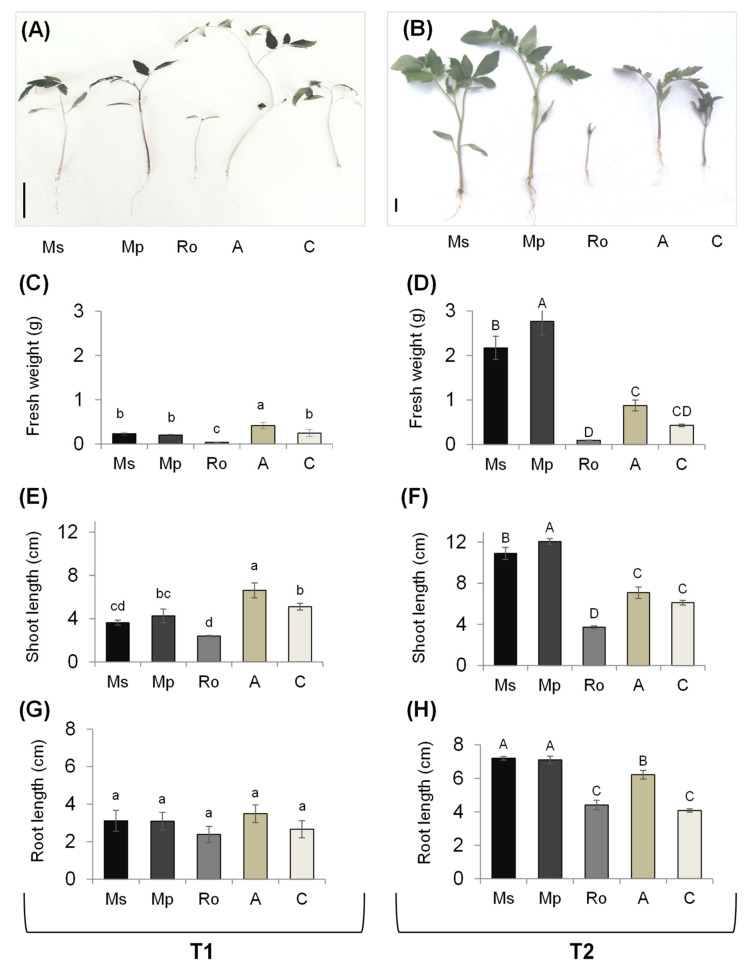
Growth indices of tomato seedlings growing in soils treated with the aromatic plants *Mentha spicata* (Ms), *M. piperita* (Mp), and *Rosmarinus officinalis* (Ro) as well as in those treated with an organic amendment (**A**) and in control soil (**C**). Measurements were taken at T1 (**A**,**C**,**E**,**G**) and T2 (**B**,**D**,**F**,**H**), 28 and 56 days, respectively, after the soil mixtures and the control were prepared, on seedlings that emerged 28 days after the tomato seeds were sown. (**A**,**B**) Representative pictures of tomato seedlings; (**C**,**D**) weights of fresh seedlings (g); (**E**,**F**) shoot lengths of fresh seedlings (cm); (**G**,**H**) root lengths of fresh seedlings (cm); the scale bar in (**A**,**B**) is equal to 2 cm. Values are averages ± SE. Different letters above the bars show significant differences among treatments within each sampling time (Duncan’s multiple range test; *p* < 0.05); values between different times were not compared.

The growth features of tomato seedlings are presented in Figure 2, with representative pictures of the seedlings from the different treatments at the two sampling times (A,B) and results regarding fresh weight (C,D), shoot length (E,F), and root length (G,H).

At T1 (Figure 2), there was no difference in the root lengths of the tomato seedlings from the different treatments. There was a difference in the shoot length, with seedlings from the Ro treatment being the shortest, and those from the organic-amendment treatment (A) being the longest; there was no difference between the control seedlings and those from either the Ms or the Mp treatment. The long seedlings from the A treatment were also the heaviest. The tomato seedlings from the Ro treatment were strikingly different from all the others: in the presence of decaying rosemary leaves, tomato seeds germinated, but seedling growth was entirely hindered afterwards. 

At T2 (Figure 2), seedlings from the Ms and Mp treatments had the longest shoots and roots and were also the heaviest of all seedlings; moreover, values for the fresh weight and shoot length were higher for the seedlings from the Mp than those from the Ms treatment. The seedlings from the Ro treatment were as they had been at T1: they were alive, but with no further growth beyond the open-cotyledon stage. In seedlings from the organic amendment treatment, the root length values were greater than those from the control. 

Although the age of seedlings was the same, their agronomic features were conspicuously different at the two sampling times, T1 and T2, which were noticeable to a larger extent in terms of the fresh weight. This can be attributed to the prevailing conditions during the two-month experimental period, which comprised colder temperatures and less sunshine in the early spring (March) than a month later. This was the case for the control and organic amendment seedlings and, to a certain extent, for the mint treatment seedlings; in the latter case, it seems that the advanced decay of the aromatic plants created a more favourable soil environment for the growth of tomato seedlings, as expressed at T2. 

### 2.3. Physiological Responses of Tomato Seedlings

**Figure 3:**
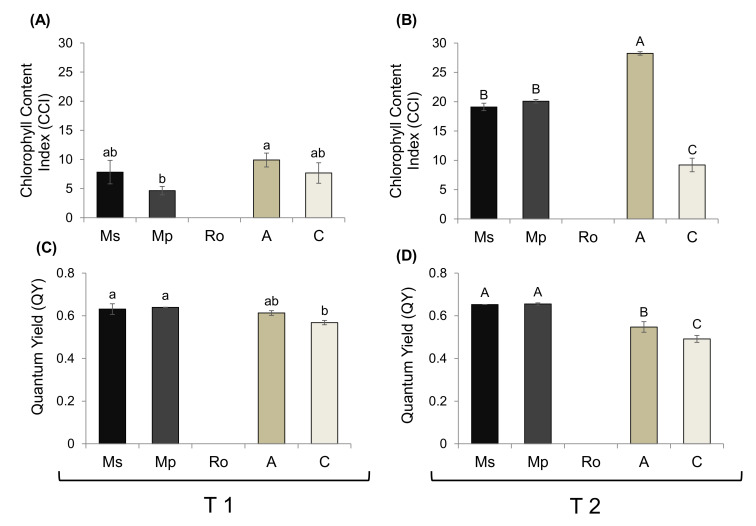
(**A**,**B**) Chlorophyll content index and (**C**,**D**) quantum yield of tomato seedlings growing in soils treated with the aromatic plants *Mentha spicata* (Ms), *M. piperita* (Mp), and *Rosmarinus officinalis* (Ro) as well as in those treated with an organic amendment (**A**) and in control soil (**C**). Measurements were taken at T1 (**A**,**C**) and T2 (**B**,**D**), 28 and 56 days, respectively, after the soil mixtures and the control were prepared, on seedlings that emerged 28 days after the tomato seeds were sown. Values are averages ± SE. Different letters above the bars (small for T1 and capital for T2) show significant differences between treatments within each sampling time (Duncan’s multiple range test; *p* < 0.05); values among different times were not compared.

The chlorophyl content index (CCI) and the photosynthetic yield (QY) of the tomato seedlings from the different treatments were estimated at T1 and T2 (Figure 3). Because the seedlings exposed to rosemary had not grown, no such measurements could be taken for them.

At T1, no difference in CCI was detected between seedlings from either the Ms or the Mp treatments and the control (Figure 3A), but at T2, their values were at least 65% higher than those of the control (Figure 3B). Nevertheless, the highest values at T2 were associated with the organic amendment treatment; they were at least 25% higher than those for the mint treatment seedlings and 70% higher than in the control. 

Regarding the photosynthetic (quantum) yield, values were higher than in the control for seedlings in the two mint treatments at both sampling times (Figure 3C,D). Seedlings of the organic amendment treatment had similar values to those of all the other treatments at T1, whereas at T2, they had intermediate values, between those in the control (low) and in the treatments with aromatic plants (high). 

### 2.4. Metabolic Responses of Tomato Seedlings

**Figure 4:**
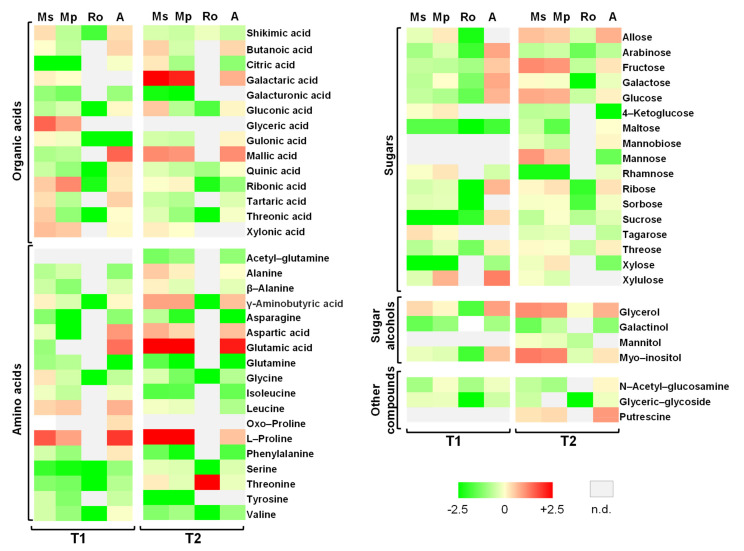
Heatmaps of metabolites indicating changes in tomato seedlings growing in soils treated with the aromatic plants *Mentha spicata* (Ms), *M. piperita* (Mp), and *Rosmarinus officinalis* (Ro) as well as in those treated with an organic amendment (A) and in control soil (C). Measurements were taken on seedlings at T1 and T2, 28 and 56 days, respectively, after the soil mixtures and the control were prepared, on seedlings that emerged 28 days after tomato seeds were sown; the values used for heatmap visualization correspond to 0.25 g of plant material. Green indicates lower, whereas red higher relative abundance; n.d. means not detected. The full colour scale is shown at the bottom of the figure.

The metabolite profiles of the tomato seedlings for the sampling times T1 and T2 are shown in Figure 4. These are heatmaps corresponding to the relative metabolite contents in the seedlings from the different treatments and their divergence from the control; the values themselves are provided in Tables S3 and S4 with the results of the statistical analyses. In total, 50 metabolites were identified at T1, whereas 52 were identified at T2. The identified compounds belong to the groups of organic acids (14 at T1; 13 at T2), amino acids (16; 16), soluble sugars (15; 16), soluble alcohols (3; 5), and other organic compounds (2; 3). In terms of the number of compounds participating in each group, the most well represented were the amino acids and the soluble sugars. 

At sampling time T1, fructose, glucose, myo-inositol, malic acid, and γ-aminobutyric acid (GABA) (Table S3) were the most abundant compounds in the seedlings, with this rank being true in almost all the treatments. These five compounds contributed always with a relative abundance above one, with the exception of myo-inositol and GABA in the Ro treatment; more specifically, the value for GABA was around 50 times lower than it was in seedlings from the other treatments, where, at maximum, the values for the other compounds were less than five times different (Table S3). The cumulative relative abundance of these five compounds was strikingly similar in seedlings from the Mp and Ms treatments. It maximized in the organic amendment treatment, followed by the control group, and minimized in the Ro treatment. Further comparisons of the metabolite profiles for the different treatments showed that only 26 compounds were identified in seedlings from the Ro treatment, where they showed their lowest values of relative abundance. Shikimic and ribonic acids, leucine, and proline had higher relative abundances in seedlings from both the Mp and Ms treatments, whereas glyceric, galactaric, tartaric and threonic acids, and xylulose only occurred in seedlings from the Ms treatment (Figure 4, Table S3). For several compounds, the highest overall relative abundances corresponded to the organic amendment seedlings. This was true for four of the most abundant compounds (fructose, glucose, myo-inositol, and malic acid) as well as for glycerol, arabinose, galactose, ribose, sucrose, threose, xylulose, leucine, proline, and aspartic, butanoic and glutamic acids; for alanine and glutamine, the lowest relative abundances were detected in seedlings from this treatment. 

The same five compounds that were the most abundant at T1 were also the most abundant at T2 (Table S4). The cumulative relative abundance of the five compounds were once again maximized in the seedlings from the Mp and Ms treatments, whereas they were minimized again in seedlings from the Ro treatment. All five compounds always contributed with a relative abundance of above one, except for GABA, which once again made a very small contribution in seedlings from the Ro treatment. At this sampling time, sucrose was also one of the most abundant compounds, with a relative abundance above one in the seedlings from all the treatments and one order of magnitude higher than it was at T1 in the seedlings from the three aromatic plant treatments. In seedlings from the Ro treatment, only very few compounds (23) could be detected: even fewer than at T1. Further comparisons of the metabolite profiles for the different treatments showed that the relative abundance of proline was at least a hundred times higher in the seedlings from the two mint treatments than those from the control and at least three times as high as the abundance in the seedlings from the organic-amendment treatment. Glycerol and glutamic acid were another two compounds with higher relative abundances in seedlings of the mint treatments compared to those from the control; additionally, for the galactaric and aspartic acids and β-alanine, values were only higher in the Ms treatment, whereas for citric acid and mannose, values were only higher in the Mp treatment. Threonine was the only compound having its highest abundance in the seedlings from the Ro treatment (Figure 4). Several metabolites were more abundant in the seedlings from the organic amendment treatment than those from the control: these are the five most abundant compounds (fructose, glucose, myo-inositol, malic acid, and GABA) as well as allose, aspartic acid, and glycerol. 

**Figure 5:**
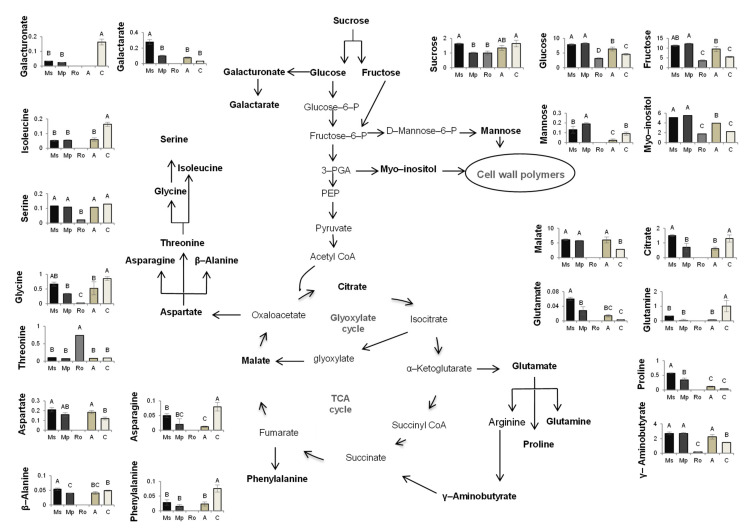
Schematic representation of metabolic pathways, with metabolites in bold representing those compounds identified in tomato seedlings growing in soils treated with the aromatic plants *Mentha spicata* (Ms), *M. piperita* (Mp), and *Rosmarinus officinalis* (Ro) as well as in those treated with an organic amendment (A) and in control soil (C). The relative abundances of the metabolites in the seedlings of the different treatments correspond to measurements at T2, 56 days after the soil mixtures and the control soil were prepared, on seedlings that emerged 28 days after tomato seeds were sown. The quantitative participation of the metabolites is expressed as relative abundance compared to the internal standard adonitol (see Table S4). Different letters above the bars show significant differences among treatments (Duncan’s multiple range test; *p* < 0.05).

A schematic representation of the metabolic pathways in which the polar metabolites that were identified play a role and which seem to be affected positively or negatively by the treatments applied is presented in Figure 5. This corresponds to T2, when the positive effects of the mint treatments on seedling growth were clearly manifested and the negative effects of the rosemary treatment continued to hold true. The relative abundance values of the metabolites that are involved in these pathways are also presented.

## 3. Discussion

It is well known that soil amendments of botanical origin can promote microbial growth by offering carbon and nutrient sources to soil microbes [32,33], provided that no inhibitory effects are exerted by the compounds that they contain. A pronounced positive effect of all three enrichments with aromatic plants was observed on all the soil microbiota examined, except for actinobacteria, which were unaffected by any of the applied treatments. Microbial biomass maximized in the spearmint and peppermint treatments, with the exception of protozoa; early in the decomposition process (at T1), their biomass took its highest value in the rosemary treatment. The absence of any effect of the organic amendment treatment on the soil microbes can be explained on the basis of the C:N ratio of the Bio-Humus organic material that we used. When an organic substrate has a low C:N ratio (up to 15), as is the case for the Bio-Humus, rapid mineralization and nitrogen release occurs, and nitrogen is available for immediate crop use. However, for microbial growth and, hence, for immobilization to occur, a C:N ratio of more than 35 is required [34]. Bacteria and fungi were the most abundant microbial groups in the soils of all treatments. These major microbial groups have been reported to be responsive also to the addition in the soil of plant material from other species like from rye and vetch [35].

A few other publications report on the effects of botanical amendments, including aromatic plants, on soil microbes [33,36,37]. It is shown [38] that soil amendments with anise, parsley, and rucola act as biostimulants, favoring soil microorganisms both in terms of biomass and functionality and that they also enhance the populations of bacterivorous nematodes, enzymatic activities, and tomato root growth. The enhancement of soil enzymatic activity and total microbial biomass by spearmint essential oil is also reported in [14]. Recently, a study on the impacts of *Thymus citriodorus* on soil communities and tomato growth showed that different plant parts had different impacts, suggesting the intervention of specific chemical constituents that were included or not in each plant part, and that soil bacterial biomass increased, whereas fungal biomass did not change in its presence [29]. In contrast, our findings showed a clear positive impact of mint and rosemary leaves on fungi, particularly at the early stages of the decomposition process.

Besides their effect on the size of microbial biomass, the spearmint and peppermint treatments also had significant impacts on the structure of the soil microbial community. Throughout the experimental period, Gram-negative bacteria were favoured over Gram-positive ones, whereas fungi were favoured over bacteria, indicating a shift in the soil microbial community towards these microbial groups from the early stages of the decomposition process. It has been demonstrated that Gram-negative bacteria are fast-growing microorganisms and respond soon after the addition of organic substrates to the soil, decrease slower than Gram-positive bacteria, and adapt better to altered environmental conditions, whereas fungi are the prime decomposers of organic materials in the soil [39]. 

The decaying green parts of aromatic plants affected the growth of tomato seedlings differently. Overall, the green parts of spearmint and peppermint had a positive effect, whereas the growth of rosemary a highly negative one. This positive effect was not expressed early in the decomposition process. In fact, at T1, leaving the seedlings from the Ro treatment aside, the only significant difference in the seedlings from the treatments with aromatic plants was in shoot length, which was lower than in the control in seedlings from the spearmint treatment. In contrast, negative effects were expressed right away; these were either temporary (e.g., shoot length in the spearmint treatment) or lasting (e.g., fresh weight in the rosemary treatment). As a result of direct nutrient release, the seedlings from the organic amendment treatment were heavier and had longer shoots than those in the control at T1 and had longer roots at T2. Different responses in plant species (maize and weeds), after incorporating aromatic plants into the soil as green manure, are also reported in [40]. 

Chlorophyll content was clearly higher than in the control at T2, in seedlings of the mint and the organic amendment treatments, with its highest value being seen in the latter. This suggests that the nutrient supply is more readily available in the case of the organic amendment treatment. The photosynthetic yield for seedlings from the mint treatments was higher than for the control ones from the start (T1); it was later (at T2) for seedlings from the organic amendment treatment. This is in accordance with the findings in [27], which showed higher photosynthetic parameter values in tomato seedlings grown in soil enriched with spearmint. Our results clearly show the absence of any inhibitory effect of spearmint and peppermint on the tomato photosynthetic apparatus. As for the rosemary treatment, no such assessment could be made because of the complete inhibition of tomato growth. 

The very similar positive impacts of the decaying green parts of spearmint and peppermint on soil microbiota, as detected by PLFA analysis, and on the growth and photosynthetic indices of tomato seedlings suggest a beneficial recruitment of soil microorganisms by mint plants and the subsequent regulation of tomato growth. This is in line with previous studies suggesting an induced by aromatic plants shift of the microbial balance in the soil towards beneficial strains or improvement in tomato performance and resistance against phytopathogenic fungal strains, such as *Fusarium* and *Verticillium*, when spearmint is used as a soil amendment [10,13,28]. 

Marked changes were also reported regarding the essential oils from decaying peppermint and spearmint shoots in the soil [30]. Patterns of change were common for both mint treatments and included a rapid drop in oil concentration and the increase of sesquiterpenoids at the expense of monoterpenoids; in fact, carvone, a major constituent of both spearmint and peppermint, almost disappeared two months after these plants were incorporated into the soil [30]. However, in vitro experiments showed carvone to be one of the most active compounds in the inhibition of seed germination and seedling growth and the spearmint essential oil to be inhibitory against various plants, including tomato [21,23]. The fact that soil amendments with spearmint have been repeatedly shown to promote tomato growth [27,28] suggests that the removal or transformation of carvone coincides with the emergence of growth-promoting factors. It has been demonstrated that the arbuscular mycorrhizal fungi colonization that is advantageous for plants is induced by sesquiterpenoids [41]. This could be an emerging growth-promoting factor in the mint treatments, given the increase of sesquiterpenoids in the essential oils of decaying mint plants, the concurrent microbial recruit, and the positive effects on soil fungi.

The results regarding seedling growth in the presence of the decaying leaves of rosemary were absolutely striking. Although there were no lethal effects, the tomato seedlings that emerged could not move beyond the open-cotyledon stage. Seedlings used the stored nutrients to sprout, but growth ended there. Exploration of the decomposition process of the hard rosemary leaves showed that rosemary oil and its individual constituents persist for a long time in the soil [30]. Under the same conditions as in the current experiments, and in contrast to peppermint and spearmint, rosemary essential oil decreased only by 50% after two months in the soil, no change in the mono- to the sesquiterpene ratio was observed, and the major constituent, cineol, continued to have high participation in the oil (>35%) [30]. This compound has been repeatedly found to have strong inhibitory activity against plant growth [21,42] and has already been evaluated as a herbicidal agent [43]. As reported in [30], this slow rate of change for the rosemary oil is accompanied by the slow decomposition rate of the rosemary leaves: even a year after their incorporation in the soil, the rosemary leaves were still discernible.

To further elucidate the impact of the different soil amendments into seedling metabolism and to detect changes that might explain the observed outcomes, i.e., growth stimulation or inhibition, polar metabolite profiling of the tomato seedlings was performed. Different metabolic responses were observed depending on treatment and sampling time, the latter being associated with different stages of decay of the aromatic plants that had been added to the soil. At T1, several metabolites that were identified in the tomato seedlings were the most abundant in the organic amendment treatment. Aspartate and glutamate that are both involved in nitrogen assimilation [44] are among these metabolites; direct nitrogen supply by the organic amendment can lead to accumulation of these two metabolites and then to growth increment. Glutamate also serves as a chlorophyll precursor [45]; hence, it can be associated with the high chlorophyll content levels that were also detected in the seedlings from this treatment. The accumulation of several sugars suggests a parallel regulation of carbon metabolism. We can therefore argue that the beneficial impact of the organic amendment on tomato growth at T1 is primarily associated with nitrogen input and to the modification of several metabolic pathways. 

Compared to control seedlings, proline was detected at higher relative abundances in seedlings from the organic amendment treatment at T1, and from the two mint treatments mainly at T2. It is well known that the accumulation of this compound in plants is strongly associated with stress conditions [46] and with priming [47]. Its presence in the seedlings of the above treatments suggests that adding aromatic plants or organic amendment to soil operates as a stress factor: only temporarily in the case of organic amendment, in a more durable way in the case of spearmint and peppermint treatments. Proline was not detected in the seedlings from the rosemary treatment. This outcome combined with the absence of the intermediates (citric and malic acids) of the tricarboxylic acid cycle (TCA) suggests the strong blockage of central metabolic pathways in the tomato seedlings that is caused by the rosemary leaves decaying in the soil. 

From Figure 5, which refers to T2 and summarizes the affected pathways and the metabolites that are involved in these pathways, it can be seen that the sugars fructose and glucose and the sugar alcohol myo-inositol obtain values higher than the control in the seedlings from the mint treatments, which are the longest and heaviest seedlings of all. These metabolites participate in crucial pathways for carbon metabolism and cell-wall biosynthesis and are classified as key metabolites for tomato growth [48]. Additionally, fructose and glucose have been proposed by several researchers as regulating growth parameters and metabolic responses [49,50,51] because of their involvement in fundamental pathways, such as the pentose–phosphate pathway and glycolysis. The molecular networks driving cell division and expansion that are essential for plant cell elongation largely rely on the availability of carbohydrates [52]. The accumulation of these metabolites, which are all precursors of cell wall components [53,54,55], in the seedlings from the mint treatments indicates the stimulation of dynamic processes, such as cell wall remodelling and/or cell proliferation. This is in line with the high levels of aspartate that support cell proliferation [56,57]. On the other hand, the low levels of galacturonic acid in the seedlings from the mint treatments suggest a possible priming state of the plants [58]. Interestingly, the decrease of galacturonic acid was accompanied by an increase of galactaric acid, as illustrated in the heatmap at T2, indicating the intensification of galacturonic acid bioconversion to galactaric acid. This reaction generates H_2_O_2_ [59] and sets oxidative conditions for the cell environment that are related to accumulation of GABA and proline [60], which are both involved in priming responses [61,62]. Overall, metabolomic analysis revealed that the plant metabolism of the seedlings from the mint treatments is activated towards two directions: growth and priming, at the same time. The contribution of the soil microbial community in these processes is not known; it is an interesting issue that needs to be addressed.

In the seedlings from the rosemary treatment, only few metabolites of low abundances were identified; this did not hold true for threonine, which obtained its highest abundance in the rosemary-treated seedlings. The accumulation of this amino acid may be related to its role as a signal molecule [63] in oxidative stress caused by biotic or abiotic factors, as suggested recently [64]. The high levels of threonine in the rosemary treatment can be attributed to its limited conversion to glycine and isoleucine (Figure 5). Given that glycine is also involved in plant photorespiration [65] and that isoleucine contributes to TCA cycle feeding, the low levels of these two compounds in this treatment indicate once again the repression of central metabolic pathways, resulting in tomato growth limitation. These findings seem to be associated with the excess of cineol, which is present for long periods of time in soils enriched with rosemary [30,66]. 

In conclusion, essential oil constituents are biologically active compounds that have both inhibitory and stimulating effects on plant and microbial growth. Their persistence in the soil environment and the changes that they undergo are crucial for their subsequent impact on plants, as they may exert their activity directly, in their original chemical form, or after they are transformed. What we see in plant growth is the result of processes that take place both within and outside the plant, where microbes play crucial roles. The cases presented here, of the two types of mint and rosemary that contain essential oils with highly active compounds against plant growth, as evidenced in laboratory experiments, correspond to two contrasting patterns. They can both find important applications in agriculture, but we need to further elucidate the underlying mechanisms resulting in suppressing or stimulating effects. In this context, we need to understand what the specific molecules involved in rhizosphere’s microbial recruitment and in the plant–microbe interactions associated with these growth outcomes are. We also note that both spearmint and peppermint are economically important herbs. Their application at a large scale, as in tomato fields, to make use of their growth-promoting properties may not be feasible because of high production costs. However, as effects are expressed early, less than a month after sowing, these plants could be applied in seedbeds for producing vigorous seedlings ready to be transplanted at an earlier time, thus satisfying a highly desirable goal of plant nursery owners. Further research should also examine their potential use in mixed cropping or crop-rotation systems.

## 4. Material and Methods

### 4.1. Plant and Soil Material

The aromatic plants that we used were peppermint (*Mentha piperita;* Mp), spearmint (*Mentha spicata;* Ms), and rosemary (*Rosmarinus officinalis;* Ro*)*; they were commercially supplied. The two *Mentha* species are perennial herbs sharing similar essential oil features, while rosemary is an evergreen shrub with an essential oil of very different content and composition. Carvone (28%) followed by menthol (23%) are the main constituents of spearmint, menthol (40%) is the main constituent of peppermint, whereas cineol (45%) is the main constituent of rosemary [30]. The plant parts used here were the whole aboveground green biomass for peppermint and spearmint and the green upper part of shoots for rosemary. The biomass was dried and coarsely chopped immediately before mixing.

The soil used for the experiments was from the Aristotle University of Thessaloniki farm, from a field left in fallow for at least 10 years. It was a silty clay loam (32% clay, 56% silt, 12% sand) with a of pH 7.8. A detailed analysis of its physicochemical properties is given in [27]. 

### 4.2. Experimental Setup

Aromatic plants were mixed with soil at a rate of 4%. Mixtures made with each one of the three aromatic plants (1.2 kg) were put in 2 kg pots. Apart from these three treatments, there were control soil samples without aromatic plants and soils treated with the commercially supplied organic amendment, Bio-Humus (pH 6.7–7.8, C/N: 12–15:1, organic carbon 26–32% *w*/*w*, organic matter 45–60%, nitrogen 1.5–2.5% *w*/*w*, and phosphorus 1–2%, according to the supplier). There were three replicate pots per treatment. The pots used in the experiment were all prepared at the same time and were placed in a greenhouse within the university farm. During the experimental period, the temperature range in the greenhouse was 16–24 °C and the relative humidity 45–60 %. A total of ten tomato seeds (*Solanum lycopersicum*, var. EZ Noam) were sown per pot at two times: (i) in one set of pots in early March, at the start of the experiment, when aromatic plants and the organic amendment were mixed with soil and (ii) in another set of pots, 28 days after the start of the experiment (T1), in early April. All the prepared pots, with or without tomato seeds sown, were watered to full capacity and then every two or three days. For both (i) and (ii), tomato and soil sampling took place four weeks later: at T1 for the pots sown at the start of the experiment and at T2, i.e., 56 days after the start of the experiment, for the pots sown at T1. This means that while emerged tomato seedlings were always analysed four weeks after the tomato seeds were sown, decaying aromatic plants, which were in the soils where the seedlings grew, were at different stages, decaying for four weeks for (i) and eight weeks for (ii). 

### 4.3. Soil Microbial Community Structure (PLFA Analysis)

To have an estimate of the structure and abundance of the soil microbial community, of the abundance of specific microbial groups, and of how these change with time and treatment, we applied phospholipid-derived fatty acid (PLFA) analysis. Sampling was conducted at T1 and T2. All samples were stored at 4 °C until analysis, and sampling was always performed within a week. The extraction and analysis of the phospholipids from the soil samples were performed according to [31] and as described in detail in [32]. Chromatographic separation and identification of the main PLFA methyl-esters were performed on Trace GC ultra-gas chromatograph (Thermo Finnigan, San Jose, CA, USA), as reported in [33]. Fatty acid methyl esters (FAME) were identified through the comparison of their relative retention times and mass spectral fragmentation patterns to those of the authentic standard mixtures using the Supelco 37-component Fatty Acid Methyl Ester (FAME) Mix and the 26-component Bacterial Acid Methyl Ester (BAME) Mix (47885-U and 47080-U, respectively; Supelco, Bellefonte, PA, USA) as well as with computerized searches against the NIST98 commercial library (National Institute of Standards and Technology, Gaithersburg, MD, USA). Quantification of each fatty acid (in nmol g^−1^) was achieved using a calibration curve constructed against the GC detector response for the internal standard 19:0 ME. Under the above-described conditions, the GC response to the 19:0 methyl ester was linear and in the range of 25–200 μg mL^−1^, with acceptable recoveries [31,33]. 

### 4.4. Measurement of Photosynthetic and Growth Parameters

The photosynthetic yield (QY) and the chlorophyll content index (CCI) were recorded in tomato seedlings, which emerged 28 days after the seeds were sown, before they were sampled for other analyses at both T1 and T2. Measurements for both physiological parameters were taken approximately three hours after sunrise and were conducted on the second fully expanded leaf below the plant apex. No measurements were performed in the tomato seedlings from the Ro treatment because their growth was entirely inhibited: they remained at the open-cotyledon stage throughout the experiment. Photosynthetic yield (effective quantum yield of photochemical energy conversion in PSII) or simply the quantum yield (QY) was recorded using a direct portable fluorometer (photosynthesis yield analyzer MINI–PAM, Walz, Effeltrich, Germany) and were computed in terms of the energy harvesting efficiency by open PSII reaction centers in the light, as previously described in [27]. The chlorophyll content index was measured using an Opti-Sciences CCM-200 chlorophyll content meter (OptiSciences Inc., Tyngsboro, MA, USA). For both QY and CCI, nine measurements per treatment (three per pot) were taken at each sampling time. 

Right after sampling for photosynthetic parameters, we recorded the shoot length, root length, and fresh weight of all the seedlings that had emerged in the pots of the different treatments. 

### 4.5. Metabolite Extraction, Derivatization, and Profiling after Gas Chromatography–Mass Spectrometry (GC–MS) Analysis

After taking growth measurements, two seedlings were removed from each replicate pot for each treatment, except for the rosemary treatment. They were immediately frozen in liquid nitrogen and stored at −80 °C for polar metabolite analysis. Of the six seedlings sampled per treatment, five were eventually analysed; these were biological replicates. In the case of the rosemary treatment, because of their very small size, four seedlings were removed from each pot, i.e., twice as many as in the other treatments and, accordingly, each of the five biological replicates was now made of two seedlings. After homogenization, 0.25 g from each sample were analysed for metabolites; whenever the seedling material was not sufficient, values were normalized to correspond to this amount before any statistical analyses and comparisons. 

The determination of the primary polar metabolites was conducted as described in [67] by GC-MS analysis following derivatization with methoxy-amine hydrochloride (Sigma Aldrich, St. Louis, MI, USA) in pyridine and N-methyl-N-trimethylsilyl-trifluoroacetamide (MSTFA reagent; Supelco Bellefonte, PA, USA). Chromatographic separation and identification of metabolites was performed on a Trace GC Ultra-Gas Chromatograph (Thermo Finnigan, San Jose, CA, USA) coupled with a Trace ISQ mass spectrometry detector, a TriPlus RSH autosampler with a split–splitless injector, and an Xcalibur MS platform. Next, 1-μL samples were injected with a split ratio of 70:1. GC separations were conducted on a 5% phenyl-methylsiloxane-fused silica capillary column (TR-5MS 30 m × 0.25 mm × 0.25 μm) with helium as a carrier gas at a flow rate of 1 mL min^−1^. The peak area integration and chromatogram visualization were performed using the Xcalibur processing program. For peak identification and mass spectra tic evaluation, the NIST11 database (National Institute of Standards and Technology, Gaithersburg, MD, USA) was used. Mass spectra were cross referenced with those of authentic standards in the Golm metabolome database (gmd.mpimp-golm.mpg.de) [67,68]. Quantification of the detected metabolites was based on comparisons with the internal standard adonitol and was expressed as relative abundances.

### 4.6. Statistics

Statistical analyses were conducted using SPSS version 17.0 For the PLFA data, two-way analysis of variance (ANOVA) was performed to determine the effect of time, treatment, and their interaction. To further explore the treatment effect for each sampling time, data were subjected to one-way ANOVA, separately for each sampling time (T1, T2) followed by Duncan’s multiple range test (*p* < 0.05), again, separately for each sampling time (T1, T2). For the photosynthetic and growth parameters, the average value per pot represented one replicate. For the metabolites, there were five samples per treatment. Data for all the examined plant parameters were subjected to one-way ANOVA followed by Duncan’s multiple range test (*p* < 0.05); the test was applied separately for each parameter and sampling time. Different letters in the graphs indicate significant differences according to Duncan’s multiple range test (*p <* 0.05); small letters correspond to sampling time T1, and capital letters correspond to T2. SE in results corresponds to standard error.

## 5. Conclusions

This multidimensional study provides insight (i) into the changes in the growth and metabolism of tomato seedlings at two different time points, after soil was enriched with *Μentha spicata, M. piperita,* and *Rosmarinus officinalis* green parts and (ii) on the impacts of these enrichments on the microbial community of the treated soils used as seedbeds. Our results highlighted the strong negative impact that rosemary had on tomato seedling growth, which was evident from the beginning to the end of the experimental period, and, in contrast, the positive impact of soil enrichments with spearmint or peppermint, which was detected with agronomic and photosynthetic measurements. They also provided insight into the metabolites that were accumulated or depleted as well as the affected metabolic pathways, which were associated with growth and priming. Results showed that enrichment with all three aromatic plants had positive impacts on the soil microbial community, increasing the biomass of all microbial groups except for the unaltered actinobacteria. In addition, they showed that enrichment with the two mints changed the structure of the soil microbial community, with Gram-negative bacteria and fungi being favoured over Gram-positive bacteria and bacteria, respectively. 

Comparing all results, we find both positive and negative effects detected on plant growth but only positive ones on the growth of soil microbes. Negative impacts on plant growth were detected early in the decomposition process of the aromatic plants incorporated in the soil, and they were long-lasting; this is the case of rosemary. Positive impacts were more accentuated early in the decomposition process for microbes but later for plants; the latter refers to spearmint and peppermint treatments. This indicates that the negative impacts on plant growth were directly induced by the compounds present in the rosemary essential oil, but this does not hold true for the positive effects of the two mints; processes mediated by microbes that lead to the transformation of compounds participating in their essential oils seem to be required for positive effects to be exerted. 

The results of this study indicate a great potential for aromatic plants to find important novel applications in agriculture. As impacts in the field may deviate substantially from our expectations that are based on laboratory experiments, many combinations of aromatic and crop plants should be evaluated to find the ones that maximize the desired outcome. 

## Data Availability

The data presented in this study are available upon request from the corresponding author, K.K.

## Supplementary Materials

The following are available online at www.mdpi.com/article/10.3390/plants10091848/s1, Table S1: Phospholipid fatty acid content of microbial origin in soils treated with the aromatic plants *Mentha spicata* (Ms), *M. piperita* (Mp), and *Rosmarinus officinalis* (Ro) as well as in those treated with an organic amendment (A) and in control soil (C). Measurements were taken at T1 (28 days after the soil mixtures and the control were prepared). Values are means of three replicates ± standard error; different letters indicate significant differences among treatments (Duncan’s multiple range test; *p* < 0.05), Table S2: Phospholipid fatty acid content of microbial origin in soils treated with the aromatic plants *Mentha spicata* (Ms), *M. piperita* (Mp), and *Rosmarinus officinalis* (Ro) as well as in those treated with an organic amendment (A) and in control soil (C). Measurements were taken at T2 (56 days after the soil mixtures and the control were prepared). Values are means of three replicates ± standard error; different letters indicate significant differences among treatments (Duncan’s multiple range test; *p* < 0.05), Table S3: GC–MS-based metabolite profiling of tomato seedlings growing in soils treated with the aromatic plants *Mentha spicata* (Ms), *M. piperita* (Mp), and *Rosmarinus officinalis* (Ro) as well as in those treated with an organic amendment (A) and in control soil (C). Measurements were taken at T1 (28 days after tomato seeds were sown and 28 days after the soil mixtures and the control were prepared). Quantities of the detected metabolites are expressed as relative abundances compared to the internal standard adonitol. Values are the means of five replicates ± standard error; different letters indicate significant differences among treatments (Duncan′s multiple range test; *p* < 0.05), Table S4: GC–MS-based metabolite profiling of tomato seedlings growing in soils treated with the aromatic plants *Mentha spicata* (Ms), *M. piperita* (Mp), and *Rosmarinus officinalis* (Ro) as well as in those treated with an organic amendment (A) and in control soil (C). Measurements were taken at T2 (28 days after tomato seeds were sown and 56 days after the soil mixtures and the control were prepared). Quantities of the detected metabolites are expressed as relative abundances compared to the internal standard adonitol. Values are the means of five replicates ± standard error; different letters indicate significant differences among treatments (Duncan′s multiple range test; *p* < 0.05).
