# Supplementary material for: Impacts of Decaying Aromatic Plants on the Soil Microbial Community and on Tomato Seedling Growth and Metabolism: Suppression or Stimulation?
Source: Plants (Basel). 2021 Sep 6;10(9):1848. doi: 10.3390/plants10091848 (PMC8471824; doi:10.3390/plants10091848)
Supplement: Supplementary file 1 [file plants-10-01848-s001.zip › plants-1331671-supdone.pdf]

SUPPLEMENT

Impacts of Decaying Aromatic Plants on the Soil Microbial Community and on Tomato Seedling Growth and Metabolism: Suppression or Stimulation?

Aggeliki Ainalidou [1], Foteini Bouzoukla [1], Urania Menkissoglu-Spiroudi [2], Despoina Vokou [3,*] and Katerina Karamanoli [1,*]

[1] Laboratory of Agricultural Chemistry, School of Agriculture, Faculty of Agriculture Forestry and Natural Environment, Aristotle University of Thessaloniki, 54124 Thessaloniki, Greece; ainalidoy_aggeliki@yahoo.gr; vouzouklafoteini@gmail.com
[2] Pesticide Science Laboratory, School of Agriculture, Faculty of Agriculture Forestry and Natural Environment, Aristotle University of Thessaloniki, 54124 Thessaloniki, Greece; rmenkis@auth.gr
[3] Department of Ecology, School of Biology, Aristotle University of Thessaloniki, 54124 Thessaloniki, Greece
*Correspondence: vokou@bio.auth.gr; katkar@agro.auth.gr

Table S1. Phospholipid fatty acid content of microbial origin in soils treated with the aromatic plants *Mentha spicata* (Ms), *M. piperita* (Mp), and *Rosmarinus officinalis* (Ro), as well as in those treated with an organic amendment (A), and in control soil (C). Measurements were taken at T1 (28 days after the soil mixtures and the control were prepared). Values[1] are means of three replicates ± standard error; different letters indicate significant differences among treatments (Duncan's multiple range test; $p<0.05$).

Lipid acids	Concentration (nmol g^{-1} soil)				
	Treatments				
	Ms	Mp	Ro	A	C
12:0	0.24±0.07 b	0.18±0.09 ab	0.20±0.06 ab	0.11±0.01 a	0.12±0.09 a
i14:0	1.69±0.60 c	1.40±0.47 bc	0.93±0.30 ab	0.43±0.09 a	0.46±0.26 a
14:0	1.89±0.53 c	1.81±0.57 bc	1.19±0.33 ab	0.67±0.10 a	0.71±0.38 a
i15:0	5.82±1.24 b	9.58±2.55 c	5.21±0.66 b	4.07±0.73 a	4.13±1.56 ab
a15:0	11.43±6.08 b	5.86±1.55 a	5.35±3.50 a	2.25±0.38 a	2.61±0.34 a
15:0	1.83±0.54 b		1.09±0.21 a	0.73±0.11 a	0.65±0.18 a
16:1ω7c	22.57±3.98 c	25.81±4.32 c	14.56±2.10 b	7.73±1.62 a	8.21±2.68 a
16:0	38.75±4.41 c	50.43±16.70 c	21.37±1.04 b	11.73±2.31 a	11.45±2.96 a
10Me 16:0	5.24±3.56 a	3.94±0.83 a	3.38±0.22 a	3.86±0.99 a	3.34±0.51 a
a17:0	3.53±2.25 b	2.71±0.68 ab	2.56±1.01 ab	1.62±1.60 a	1.80±1.10 a
17:0	5.43±1.55 c	5.49±1.32 c	3.94±0.38 bc	2.88±0.74 a	3.48±1.30 ab
10Me 17:0	0.89±0.57 a	0.76±0.28 a	0.60±0.06 a	0.44±0.09 a	0.46±0.05 a
18:2ω6,9c		21.73±1.73 c	8.98±0.77 b	3.46±2.45 a	1.46±1.01 a
18:1ω9c	0.62±0.52 a	14.85±13.24 d	13.67±1.57 d	6.18±1.35 c	3.66±2.11 b
18:2ω3,9c	24.43±22.16 a	45.60±1.89 a			
18:1ω9t	30.93±16.60 c	17.84±1.87 b	14.42±2.53 b	6.92±2.30 a	7.40±0.67 ab
18:3ω3,6,9c	11.69±5.77 b	5.23±9.06 ab	0.47±0.45 a	0.57±0.03 a	2.92±2.88 a
18:1ω5c	0.81±1.40 a			1.11±1.08 a	1.39±0.58 a
18:0	8.44±1.46 c	7.24±2.27 c	4.39±0.78 b	1.50±0.89 a	1.56±1.16 a
11Me 18:1ω6	1.51±0.64 cd	1.71±0.33 d	1.06±0.30 bc	0.71±0.51 ab	0.45±0.19 a
10Me 18:0	2.96±1.03 b	3.01±0.58 b	2.01±0.19 ab	1.33±0.56 a	1.69±0.42 a
cy 17:0	0.86±0.39 a	1.02±0.16 a	0.60±0.10 a	1.70±1.68 a	0.75±0.60 a
20:4ω6,9,12,15c	0.67±0.64 a		1.16±0.12 a		
20:5ω3,6,9,12,15c	0.82±0.07 b	0.82±0.14 b	0.41±0.06 a	0.21±0.05 a	0.24±0.07 a
20:0	1.55±0.52 b	1.69±0.54 b	0.86±0.09 a	0.34±0.02 a	0.38±0.08 a
22:0	1.18±0.13 b	1.49±0.48 b	0.48±0.09 a	0.30±0.06 a	0.28±0.01 a
23:0	0.24±0.05 bc	0.37±0.13 c	0.12±0.03 ab	0.10±0.02 a	0.34±0.07 bc
24:0	0.43±0.15 bc	0.56±0.14 c	0.27±0.11 ab	0.14±0.12 a	0.14±0.02 a

[1] Empty spaces mean that the specific lipid acids were not detected in the seedlings of the treatment.

Table S2. Phospholipid fatty acid content of microbial origin in soils treated with the aromatic plants *Mentha spicata* (Ms), *M. piperita* (Mp), and *Rosmarinus officinalis* (Ro), as well as in those treated with an organic amendment (A), and in control soil (C). Measurements were taken at T2 (56 days after the soil mixtures and the control were prepared). Values[1] are means of three replicates ± standard error; different letters indicate significant differences among treatments (Duncan's multiple range test; $p<0.05$).

Lipid acids	Concentration (nmol g^{-1} soil)				
	Treatments				
	Ms	Mp	Ro	A	C
12:0	0.24±0.04 a	0.19±0.07 a	0.19±0.07 a	0.23±0.14 a	0.18±0.03 a
i14:0	0.88±0.26 ab	0.90±0.30 b	0.93±0.21 b	0.58±0.15 ab	0.36±0.19 a
14:0	1.43±0.22 bc	1.64±0.74 c	1.23±0.26 abc	0.89±0.22 ab	0.56±0.31 a
i15:0	6.25±1.48 b	6.53±2.10 b	5.95±0.83 ab	5.38±1.13 ab	3.46±1.69 a
a15:0	4.36±1.13 b	4.36±1.41 b	3.72±0.58 ab	2.97±0.66 ab	1.98±1.04 a
15:0	1.20±0.45 bc	1.44±0.60 c	1.02±0.22 abc	0.74±0.11 ab	0.41±0.22 a
16:1ω7c	17.44±4.06 c	19.03±4.28 c	14.41±1.73 bc	9.47±2.12 ab	6.48±4.05 a
16:0	21.38±3.85 bc	25.44±7.89 c	18.16±2.37 bc	14.49±2.16 ab	9.54±4.60 a
10Me 16:0	4.23±0.97 ab	4.21±0.94 ab	4.40±0.42 b	4.40±0.76 b	2.87±1.45 a
a17:0	3.10±0.83 ab	3.37±1.29 b	3.13±0.52 ab	2.81±0.60 ab	1.74±0.99 a
17:0	4.56±1.33 ab	5.09±1.70 b	4.17±0.54 ab	4.65±1.08 ab	2.81±1.50 a
10Me 17:0	0.67±0.19 b	0.64±0.26 ab	0.58±0.06 ab	0.54±0.09 ab	0.38±0.15 a
18:2ω6,9c	10.77±2.77 bc	14.07±4.11 c	8.31±0.24 b	4.00±1.43 a	3.42±1.67 a
18:1ω9c			10.79±1.02 b	7.60±1.83 a	6.12±2.96 a
18:2ω3,9c	15.93±3 a	21.23±5.09 b			
18:1ω9t	10.9±1.39 b	10.19±1.23 b	12.16±0.75 b	9.15±1.92 b	4.85±4.34 a
18:0	4.84±1.24 bc	5.76±0.99 c	4.93±1.15 bc	3.60±0.49 ab	2.91±1.02 a
11Me 18:1ω6	1.05±0.33 ab	1.20±0.50 b	1.09±0.21 ab	0.52±0.08 a	0.80±0.65 ab
10Me 18:0	2.30±0.6 a	2.30±0.73 a	2.03±0.27 a	2.12±0.33 a	1.35±0.72 a
cy 17:0	0.71±0.14 b	0.75±0.27 b	0.54±0.02 ab	0.50±0.10 ab	0.34±0.23 a
20:4ω6,9,12,15c	0.97±0.19 b	0.98±0.11 b	1.15±0.13 b	0.44±0.06 a	0.31±0.20 a
20:5ω3,6,9,12,15c	0.64±0.06 c	0.51±0.11 bc	0.30±0.09 ab	0.19±0.07 a	0.13±0.12 a
20:0	1.02±0.49 b	1.46±0.65 c	0.81±0.16 ab	0.59±0.17 ab	0.35±0.22 a
22:0	0.80±0.45 ab	1.19±0.63 b	0.50±0.11 a	0.51±0.12 a	0.29±0.17 a
23:0	0.15±0.08 ab	0.24±0.13 b	0.15±0.03 ab	0.15±0.02 ab	0.08±0.06 a
24:0	0.26±0.11 ab	0.36±0.18 b	0.30±0.06 ab	0.31±0.07 ab	0.17±0.12 a

[1] Empty spaces mean that the specific lipid acids were not detected in the seedlings of the treatment.

Table S3. GC–MS–based metabolite profiling of tomato seedlings growing in soils treated with the aromatic plants *Mentha spicata* (Ms), *M. piperita* (Mp), and *Rosmarinus officinalis* (Ro), as well as in those treated with an organic amendment (A), and in control soil (C). Measurements were taken at T1 (28 days after tomato seeds were sown and 28 days after the soil mixtures and the control were prepared). Quantities of the metabolites detected are expressed as relative abundances compared the internal standard adonitol. Values[1] are means of five replicates ± standard error; different letters indicate significant differences among treatments (Duncan's multiple range test; p<0.05).

**Metabolites**	**Relative abundance**				
	**Treatments**				
	**Ms**	**Mp**	**Ro**	**A**	**C**
Organic acids					
Shikimic	0.105 d<br>±0.011	0.054 b<br>±0.005	0.024 a<br>±0.006	0.105 d<br>±0.009	0.084 c<br>±0.008
Butanoic	0.009 b<br>±0.001	0.006 a<br>±0.001		0.012 c<br>±0.001	0.009 b<br>±0.001
Citric	0.028 a<br>±0.003	0.018 a<br>±0.004		0.175 b<br>±0.025	0.183 b<br>±0.045
Galactaric	0.022 a<br>±0.002	0.020 a<br>±0.003			0.020 a<br>±0.007
Galacturonic	0.026 a<br>±0.003	0.021 a<br>±0.006		0.027 a<br>±0.003	0.053 b<br>±0.006
Gluconic	0.065 b<br>±0.014	0.080 bc<br>±0.012	0.014 a<br>±0.001	0.109 d<br>±0.011	0.104 cd<br>±0.011
Glyceric	0.023 a<br>±0.006	0.015 b<br>±0.002			0.008 b<br>±0.001
Gulonic	0.048 a<br>±0.008	0.042 a<br>±0.004	0.005 b<br>±0.001		0.046 a<br>±0.003
Malic	1.058 a<br>±0.221	1.157 a<br>±0.587		5.296 c<br>±0.142	1.845 b<br>±0.011
Quinic	0.308 b<br>±0.040	0.220 b<br>±0.015	0.076 a<br>0.006	0.533 c<br>±0.039	0.444 c<br>±0.052
Ribonic	0.047 c<br>±0.008	0.074 d<br>0.009	0.007 a<br>±0.001	0.038 bc<br>±0.001	0.033 b<br>±0.004
Tartaric	0.001 b<br>±0.002	0.005 a<br>±0.001		0.011 b<br>±0.001	0.008 ab<br>±0.001
Threonic	0.178 c<br>±0.037	0.060 b<br>±0.011	0.004 a<br>±0.001	0.127 c<br>±0.019	0.125 c<br>±0.011
Xylonic acid	0.036 a<br>±0.006	0.033 a<br>±0.005		0.024 a<br>±0.002	0.023 a<br>±0.003
Amino acids					
Alanine	0.036 b<br>±0.007	0.044 b<br>±0.004		0.028 a<br>±0.005	0.059 c<br>±0.004
Asparagine	0.024 b<br>±0.004	0.005 a<br>±0.001		0.025 b<br>±0.005	0.054 c<br>±0.017
Aspartic acid	0.049 a<br>±0.011	0.005 ab<br>±0.001		0.116 c<br>±0.022	0.057 b<br>±0.001
γ-Aminobutyric acid	1.397 c<br>±0.153	0.999 b<br>±0.086	0.035 a<br>±0.004	1.342 c<br>±0.041	1.276 bc<br>±0.165

Glutamic acid	0.003 a ±0.001			0.016 c ±0.002	0.006 b ±0.001
Glutamine	0.018 b ±0.001	0.021 ab ±0.007			0.034 a ±0.006
Glycine	0.594 d ±0.032	0.371 bc ±0.078	0.009 a ±0.002	0.326 b ±0.051	0.496 cd ±0.041
Isoleucine	0.069 ab ±0.005	0.051 a ±0.008		0.070 ab ±0.009	0.077 b ±0.009
Leucine	0.052 b ±0.008	0.057 bc ±0.004	0.048 a ±0.001	0.066 c ±0.003	0.039 a ±0.007
Phenylalanine	0.018 ab ±0.001	0.012 a ±0.004		0.028 b ±0.006	0.024 b ±0.003
Proline	0.078 bc ±0.022	0.047 ab ±0.009		0.097 c ±0.015	0.025 a ±0.02
Serine	0.044 b ±0.005	0.034 b ±0.004	0.007 a ±0.001	0.084 c ±0.009	0.170 d ±0.023
Threonine	0.062 b ±0.015	0.056 b ±0.012	0.007 a ±0.001	0.086 b ±0.005	0.141 c ±0.022
Tyrosine	0.017 ab ±0.003	0.010 a ±0.002		0.016 ab ±0.002	0.023 b ±0.005
Valine	0.052 c ±0.003	0.035 b ±0.001	0.006 a ±0.001	0.068 d ±0.007	0.070 d ±0.008
β- Alanine	0.017 ab ±0.002	0.011 a ±0.002		0.019 bc ±0.003	0.024 c ±0.002
Soluble sugars					
Allose	0.022 a ±0.002	0.016 b ±0.001	0.004 c ±0.001		0.019 b ±0.001
Arabinose	0.056 bc ±0.009	0.039 ab ±0.005	0.019 a ±0.002	0.130 d ±0.013	0.071 c ±0.005
Fructose	4.440 a ±0.442	4.619 a ±0.473	3.750 a ±0.165	9.807 c ±0.415	6.832 b ±0.456
Galactose	0.151 b ±0.026	0.095 a ±0.015	0.063 a ±0.013	0.269 c ±0.029	0.150 b ±0.007
Glucose	2.576 b ±0.089	2.954 b ±0.269	1.555 a ±0.073	7.382 d ±0.611	4.435 c ±0.331
4-Ketoglucose	0.034 a ±0.005	0.029 a ±0.002			0.030 a ±0.004
Maltose	0.035 b ±0.006	0.035 b ±0.005	0.017 a ±0.003	0.030 b ±0.003	0.109 c ±0.006
Rhamnose	0.032 a ±0.007	0.026 a ±0.007		0.020 a ±0.003	0.027 a ±0.005
Ribose	0.099 bc ±0.013	0.090 b ±0.008	0.018 a ±0.001	0.197 d ±0.007	0.120 c ±0.008
Sorbose	0.122 a ±0.015	0.117 a ±0.015	0.025 b ±0.004		0.144 a ±0.013
Sucrose	0.137 a ±0.028	0.182 a ±0.026	0.264 a ±0.031	1.332 c ±0.149	1.044 b ±0.157

Tagatose	0.022 a ±0.001	0.026 a ±0.004			0.021 a ±0.002
Threose	0.298 bc ±0.033	0.219 ab ±0.010	0.143 a ±0.008	0.403 d ±0.036	0.358 c ±0.044
Xylose	0.003 a ±0.001	0.007 a ±0.001		0.027 b ±0.005	0.051 c ±0.004
Xylulose	0.025 a ±0.005	0.012 b ±0.001		0.036 c ±0.007	0.015 b ±0.002
Sugar alcohols					
Galactinol	0.057 ab ±0.007	0.041 a ±0.006		0.062 b ±0.004	0.115 c ±0.012
Glycerol	0.330 b ±0.050	0.403 b ±0.053	0.093 a ±0.004	0.593 c ±0.018	0.311 b ±0.028
Myo-inositol	1.694 b ±0.161	1.822 bc ±0.073	0.583 a ±0.067	3.175 d ±0.0228	2.104 c ±0.193
Other organic compounds					
Glyceric-glycoside	0.241 b ±0.051	0.236 b ±0.034	0.026 a ±0.006	0.198 b ±0.019	0.276 b ±0.025
N-acetyl-glucosamine	0.085 b ±0.010	0.048 a ±0.004	0.049 a ±0.008	0.062 ab ±0.012	0.089 b ±0.021

[1] Empty spaces mean that the specific metabolites were not detected in the seedlings of the treatment.

Table S4. GC–MS–based metabolite profiling of tomato seedlings growing in soils treated with the aromatic plants *Mentha spicata* (Ms), *M. piperita* (Mp), and *Rosmarinus officinalis* (Ro), as well as in those treated with an organic amendment (A), and in control soil (C). Measurements were taken at T2 (28 days after tomato seeds were sown and 56 days after the soil mixtures and the control were prepared). Quantities of the metabolites detected are expressed as relative abundances compared the internal standard adonitol. Values[1] are means of five replicates ± standard error; different letters indicate significant differences among treatments (Duncan's multiple range test; p<0.05).

Metabolites	Relative abundance				
	Treatments				
	Ms	Mp	Ro	A	C
Organic acids					
Butanoic	0.011 a	0.006 b		0.010 a	0.008 b
	±0.001	±0.001		±0.001	±0.001
Citric	1.515 b	0.717 a		0.609 a	1.300 b
	±0.251	±0.063		±0.101	±0.220
Galactaric	0.279 b	0.099 a		0.077 a	0.031 a
	±0.062	±0,018		±0.017	±0.001
Galacturonic acid	0.033 b	0.024 b			0.164 a
	±0.007	±0.03			±0.023
Gluconic	0.188 c	0.080 ab	0.039 a	0.138 bc	0.129 bc
	±0.040	±0.009	±0.006	±0.031	±0.022
Gulonic	0.056 a	0.050a		0.071 b	0.066 b
	±0.002	±0.002		±0.007	±0.004
Malic	6.094 b	5.692 b		6.024 b	2.765 a
	±0.159	±0.257		±1.008	±0.100
Quinic	0.642 b	0.532 a	0.428 a	0.775 c	0.789 c
	±0.066	±0.038	±0.019	±0.032	±0.044
Ribonic	0.064 b	0.070 b	0.014 a	0.018 a	0.065 b
	±0.009	±0.006	±0.002	±0.008	±0.015
Shikimic	0.112 a	0.110 a	0.138 ab	0.109 a	0.157 b
	±0.011	±0.011	±0.007	±0.011	±0.009
Tartaric	0.014 b	0.012 b		0.015 ab	0.020 a
	±0.001	±0.001		±0.003	±0.001
Threonic	0.170 c	0.111 b	0.040 a	0.207cd	0.220 d
	±0.008	±0.007	±0.005	±0.017	±0.022
Xylonic	0.027 a	0.024 a			0.024 a
Amino acids					
Acetyl-glutamine	0.049 a	0.061 a		0.058a	0.122 b
	±0.007	±0.003		±0.004	±0.016
Alanine	0.063 a	0.049 a		0.044 a	0.044 a
	±0.015	±0.009		±0.006	±0.005
β-Alanine	0.054 c	0.040 a		0.041ab	0.049 b
	±0.001	±0.003		±0.006	±0.001
γ-Aminobutyric acid	2.726 c	2.722 c	0.179 a	2.240 c	1.461 b
	±0.136	±0.176	±0.009	±0.309	±0.047
Asparagine	0.051 a	0.019 a		0.012 a	0.080 b
	±0.017	±0.006		±0.002	±0.013
Aspartic acid	0.207 b	0.159 ab		0.185 b	0.121 a

	±0.013	±0.020		±0.016	±0.014
Glutamic acid	0.060 c ±0.009	0.028 b ±0.004		0.014 ab ±0.003	0.003 a ±0.001
Glutamine	0.334 a ±0.076	0.035 a ±0.004		0.072 a ±0.025	1.013 b ±0.350
Glycine	0.667 bc ±0.014	0.344 b ±0.062	0.023 a ±0.002	0.522 b ±0.220	0.854 c ±0.058
Isoleucine	0.052 a ±0.004	0.054 a ±0.003		0.059 a ±0.011	0.164 b ±0.012
Leucine	0.051 ab ±0.006	0.048 ab ±0.004		0.032 a ±0.004	0.055 b ±0.005
Phenylalanine	0.028 a ±0.004	0.015 a ±0.001		0.023 a ±0.004	0.076 b ±0.020
Proline	0.573 c ±0.059	0.337 b ±0.013		0.112 a ±0.021	0.038 a ±0.009
Serine	0.116 b ±0.008	0.109 b ±0.013	0.022 a ±0.003	0.108 b ±0.021	0.138 b ±0.006
Threonine	0.103 a ±0.010	0.075 a ±0.012	0.739 b ±0.036	0.079 a ±0.019	0.091 a ±0.005
Valine	0.049 b ±0.004	0.062 c ±0.005	0.012 a ±0.003	0.058 bc ±0.004	0.113 d ±0.007
Soluble sugars					
Allose	0.024 abc ±0.002	0.026 bc ±0.003	0.013 a ±0.001	0.029 c ±0.004	0.017 ab ±0.002
Arabinose	0.067 b ±0.003	0.060 b ±0.004	0.035 a ±0.002	0.060 b ±0.005	0.094 c ±0.005
Fructose	11.245 bc ±0.304	12.203 c ±0.348	3.583 a ±0.367	9.528 b ±1.303	5.488 a ±0.269
Galactose	0.268 b ±0.040	0.269 b ±0.033	0.044 a ±0.005	0.245 b ±0.033	0.280 b ±0.039
Glucose	7.847 d ±0.331	8.211 d ±0.324	3.151 a ±0.204	6.364 c ±0.669	4.569 b ±0.270
4-ketoglucose	0.268 b ±0.019	0.257 b ±0.022		0.046 a ±0.011	0.404 c ±0.009
Maltose	0.021 a ±0.003	0.045 a ±0.010		0.064 a ±0.029	0.064 a ±0.006
Mannobiose	0.023 a ±0.001	0.029 ab ±0.002		0.0374 b ±0.010	0.035 ab ±0.003
Mannose	0.133 b ±0.011	0.186 c ±0.032		0.022 a ±0.014	0.092 b ±0.016
Ribose	0.240 b ±0.031	0.209 b ±0.025	0.050 a ±0.002	0.243 b ±0.032	0.198 b ±0.033
Sorbose	0.191 b ±0.016	0.188 b ±0.012	0.058 a ±0.002	0.182 b ±0.030	0.197 b ±0.017
Sucrose	1.618 b ±0.055	1.066 a ±0.054	1.021 a ±0.120	1.344 ab ±0.163	1.654 b ±0.205

Tagatose	0.037 b	0.035 ab		0.028 a	0.044 c
	±0.002	±0.003		±0.001	±0.021
Threose	0.238 b	0.252 b	0.169 a	0.272 b	
	±0.006	±0.008	±0.14	±0.019	0.244 b
Xylose	0.046b	0.042 b		0.019 a	0.047 b
	±0.009	±0.003		±0.005	±0.010
Xylulose	0.032 a	0.039 a			0.041 a
	±0.005	±0.006			±0.008
Sugar alcohols					
Galactinol	0.128 a	0.071 a		0.089 a	0.192 b
	±0.017	±0.010		±0.027	±0.016
Glycerol	0.674 bc	0.724 c	0.321 a	0.563 b	0.332 a
	±0.011	±0.062	±0.022	±0.047	±0.023
Mannitol	0.257 b	0.283 b	0.192 a		0.302 b
	±0.028	±0.027	±0.016		±0.010
Myo-inositol	5.085 c	5.526 c	1.771 a	3.958 b	2.227 a
	±0.190	±0.277	±0.282	±0.628	±0.088
Other organic compounds					
Glyceryl-glycoside	0.258 b	0.316 bc	0.059 a	0.399 bc	0.448 c
	±0.007	±0.038	±0.012	±0.079	±0.100
N-acetyl-glucosamine	0.040 a	0.048 ab		0.077 b	0.073 b
	±0.004	±0.003		±0.010	±0.001
Putrescine	0.031 a	0.029 a		0.048 a	0.024 a
	±0.003	±0.003		±0.022	±0.002

[1] Empty spaces mean that the specific metabolites were not detected in the seedlings of the treatment.
